# Supplementary material for: Suppression of ERK signalling promotes pluripotent epiblast in the human blastocyst
Source: Nat Commun. 2025 Jul 28;16:6922. doi: 10.1038/s41467-025-61830-x (PMC12304225; doi:10.1038/s41467-025-61830-x)
Supplement: Supplementary file 1 — Supplementary Information [file 41467_2025_61830_MOESM1_ESM.pdf]

Supplementary Data

Suppression of ERK signalling promotes pluripotent epiblast in the human blastocyst

Claire S. Simon, Afshan McCarthy, Laura Woods, Desislava Staneva, Martin Proks, Nazmus Salehin, Georgia Lea, Qiulin Huang, Madeleine Linneberg-Agerholm, Alex Faulkner, Athanasios Papathanasiou, Kay Elder, Phil Snell, Leila Christie, Patricia Garcia, Valerie Shaikly, Mohamed Taranissi, Meenakshi Choudhary, Mary Herbert, Courtney W. Hanna, Joshua M. Brickman, Kathy K. Niakan

The PDF file includes:

Supplementary Materials and Methods
Supplementary Figures. 1 to 5
Supplementary Table 3 to 4
Supplementary References

Other Supplementary Materials for this manuscript include the following:

Supplementary Tables 1 to 2 which are provided as excel sheets

Supplementary Material and Methods

Mouse embryo culture

All animal research was performed in accordance with the UK Home Office regulations under project licence PP8826065, which passed ethical review by the Francis Crick Institute Animal Welfare Review Board in 2019. Mice were maintained on a 12 h light–dark cycle, ambient temperature 19/22°C, and humidity 45/65%. Three- to -four to eight-week-old (C57BL6 × CBA) F1 female mice were super-ovulated using injection of 5 IU of pregnant mare serum gonadotrophin (PMSG; Sigma-Aldrich). Forty-eight hours after PMSG injection, 5 IU of human chorionic gonadotrophin (HCG; Sigma-Aldrich) was administered. Superovulated females were set up for mating with eight-week-old or older (C57BL6 × CBA) F1 males.

Mouse zygotes were isolated in FHM under mineral oil and cumulus cells were removed with hyaluronidase (Sigma-Aldrich; H4272). Mouse embryos were cultured in pre-equilibrated Global Media (Cooper Surgical) supplemented with 10% Human Serum Albumin (HSA, Cooper Surgical) overlaid with mineral oil (Cooper Surgical). For FGF treatments, embryos were cultured with 1000ng/ml FGF4 (R&D) and 1000ng/ml Heparin (Sigma) or in control 10% HSA, Global media; for ERKi treatments in 5µM Ulixertinib (Cambridge Bioscience); or 0.1% DMSO in 10% HSA, Global media, for 24 hours (E3.25 – E4.25) or 48 hours (E2.5-E4.5) as indicated.

Rat embryo culture

Super-ovulated and mated 7- to 9-week-old Sprague Dawley females were purchased by Charles River UK Limited. Rat two-cell embryos were isolated in FHM and then cultured in drops of pre-equilibrated rat embryo culture medium (mR1ECM, Cosmo Bio Co, CSR-RN174) and overlaid with mineral oil. Embryos were incubated at 37°C and 5% CO_2 and cultured until the day of analysis. For FGF treatments, embryos were cultured with 1000ng/ml FGF4 (R&D) and 1000ng/ml Heparin (Sigma) or in control mR1ECM medium.

Bovine IVF and culture

Oocytes were collected from bovine ovaries and matured overnight in BO-IVM medium (IVF Bioscience) at 38.5°C under normoxia. Frozen bull sperm straws (UK Sire Services) were thawed for 1min at 37°C, then transferred to BO-SEMENPREP (IVF Bioscience) followed by two rounds of washes and centrifugation at 300g for 5min. Granulosa cells were removed from cumulus-oocyte complexes (COCs) by pipetting and combined with $4x10^6$ spermatozoa per 1ml of BO-IVF media (IVF Bioscience) for 6-10hrs at 38.5°C under normoxia. COCs were then denuded and transferred to BO-IVC media (IVF Bioscience) overlayed with mineral oil with 20-30 presumptive embryos per 500µL drop followed by culture at 38.5°C under hypoxia. At 7dpf, the embryos were transferred to BO-IVC media supplemented with either 5µM Ulixertinib, 750ng/mL FGF4 and 1µg/mL Heparin or in control BO-IVC media and cultured for an additional 48hrs.

Immunofluorescence of bovine embryos

Bovine blastocysts were fixed with 100mM Hepes pH 7, 50mM EGTA pH 7, 10mM $MgSO_4$, 4% methanol-free formaldehyde and 0.2% Triton X-100 in sterile H_2O for 30min at 37°C followed by 3x washes with PBS with 3mg/mL polyvinylpyrrolidone (PVP). Fixed blastocysts were permeabilised with 0.5% Triton X-100 in PBS with 3mg/mL PVP and blocked with 3% donkey serum, 0.1% bovine serum albumin and 0.2% Triton X-100 in PBS with 3mg/mL PVP, both overnight at 4°C. Primary antibodies were diluted in blocking buffer and incubated overnight at 4°C and secondary antibodies were diluted 1:500 in blocking buffer and incubated for 2hrs at room temperature shaking. The embryos were washed 3x for 10min following each antibody incubation. DAPI was added to blocking buffer during the wash steps after secondary antibody incubation for nuclear stain. Bovine blastocysts were then imaged in blocking buffer without DAPI in 18-well ibidi slides using a Leica SP8 confocal microscope.

Human primed ESC culture

Primed H9 human ESC were cultured according in mTeSR1 (StemCell Technologies) according to the manufacturer's guidance, on Matrigel (356231, BD Bioscience) coated plates at 37°C 5% CO_2 under normoxia. H9 hESCs were dissociated using ReLeSR (Stem Cell Technologies) incubate for 4 min at 37°C and passaged in clumps. Primed hESC (seeded at 1:10) were grown in μ-Slide 8 well high chambered coverslip (Ibidi, 80806) for 48 hrs then cultured for 24 hrs in 1 μM PD0325901 (Cambridge Bioscience; 13034-1mg-CAY) or in DMSO control medium prior to fixation and immunostaining.

Human ESC karyotyping

To determine chromosome copy number, 2×10^5 cells from each of the hESC lines derived in this study were collected. To extract DNA the DNeasy® Blood & Tissue Kit was used according to the manufacture's protocol (Qiagen, 69504 and 69506). This was followed by low-pass next generation sequencing (depth of sequencing < 0.1x). Libraries were prepared using the VeriSeq PGS Kit (Illumina) or the NEB Ultra II FS Kit according to the manufacturer's instructions. The MiSeq platform or the Illumina HiSeq 4000 platforms were used. Reads were aligned to the human genome hg19 using BWA v0.7.17[1] and the copy number profiles generated with QDNaseq v1.24.0 as described previously[2].

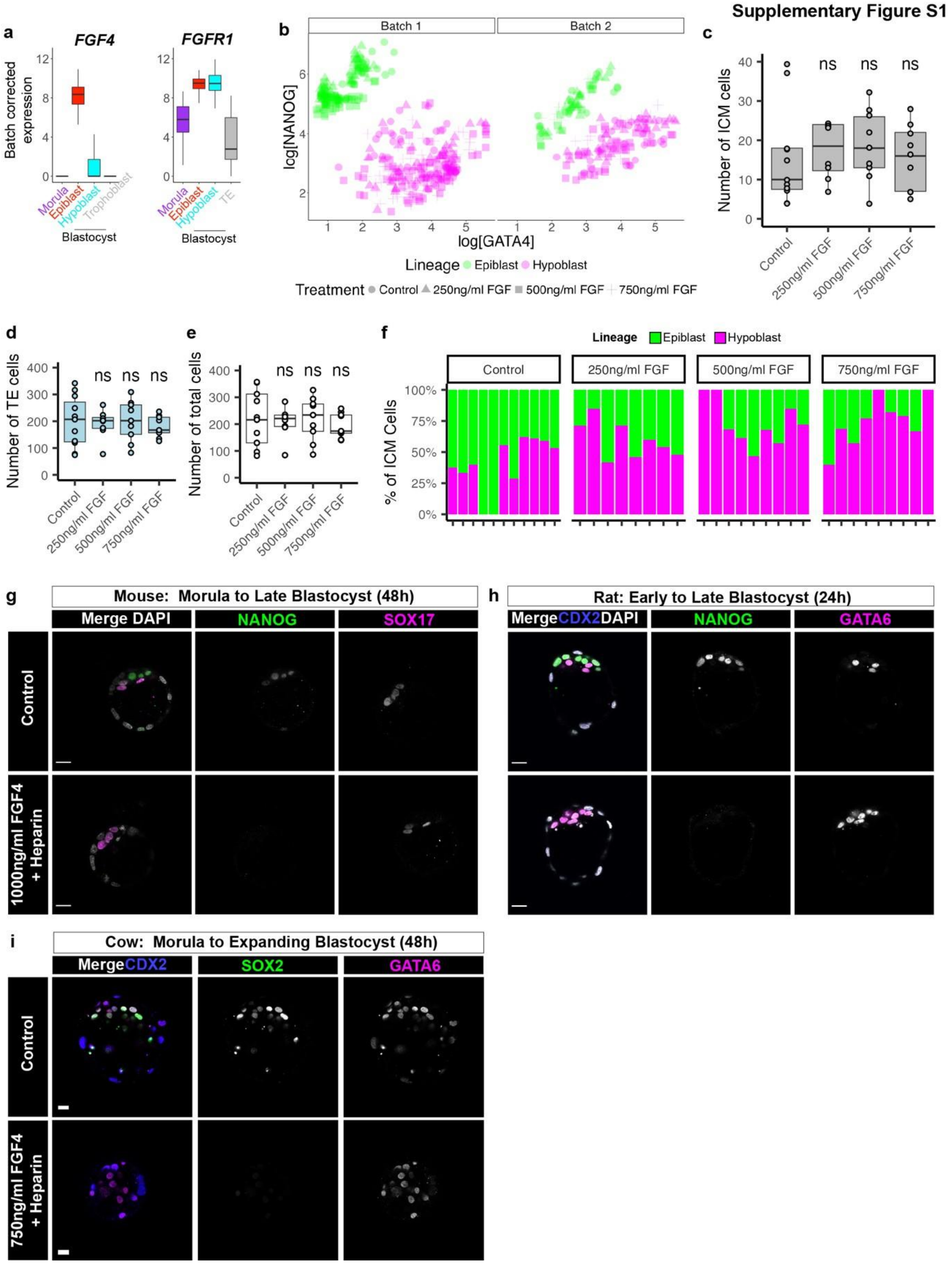

Supplementary Figure S1. Exogenous FGF is sufficient to drive human hypoblast specification.

(a) Expression of *FGF4* (ligand) and *FGFR1* (receptor) mRNA in human morula and blastocyst stage embryos in batch-corrected analysis[3] of single-cell RNAseq datasets[4-6]. **(b)** Scatter plot of NANOG and GATA4 nuclear fluorescence intensity of confocal images in Fig. 1a. Hierarchical clustering was used to assign cell lineages. Epiblast ($NANOG^{hi}GATA4^{lo}$) and Hypoblast ($NANOG^{lo}GATA4^{hi}$). Batches include experiments performed in the initial submission (Batch 1) and in revision (Batch 2) after replacement of confocal laser lines.

(c-e) Boxplots showing **(c)** the number of inner cell mass (ICM) cells (GATA3-) cells **(d)** the number of trophectoderm (TE, GATA3+) cells and **(e)** the number of total cells in human embryos cultured in increasing concentrations of FGF and Heparin. Boxplots represent the interquartile (IQR range), with the median shown as a central line; whiskers extend to lowest or highest value within 1.5*IQR; values for each embryo are shown as individual points. Comparisons to Controls were not significant $p > 0.05$, t-test.

(f) Stacked bar charts showing the proportion of epiblast and hypoblast in the ICM of individual embryos, grouped by treatment. Embryos are ordered left to right on the x-axis in ascending order of total number of cells. Control n = 11, 250ng/ml n = 8, 500ng/ml n = 9, 750ng/ml n=9.

(g) Confocal images of mouse embryos following either 1000ng/ml FGF4 plus Heparin or control treatment from day 2.5 morula stage for 48 hrs until day 4.5 late blastocyst stage. Mouse embryos were immunostained for lineage markers Nanog (epiblast), Sox17 (hypoblast), and stained for nuclear DAPI. Scale bars 20 µm. Control n = 6, FGF n = 10. **(h)** Confocal images of rat embryos following either 1000ng/ml FGF4 plus Heparin or control treatment from day 3.5 early blastocyst stage for 24 hrs until day 4.5 late blastocyst stage. Rat embryos were immunostained for lineage markers Nanog (epiblast), Gata6 (hypoblast), or Cdx2 (trophectoderm) and stained for nuclear DAPI. Scale bars 20 µm. Control n = 10, FGF n = 10.

(i) Confocal images of cow embryos following either 750ng/ml FGF4 plus Heparin or control treatment from day 7 morula stage for 48 hrs until day 9 blastocyst stage. Cow embryos were immunostained for lineage markers Sox2 (epiblast), Gata6 (hypoblast), or Cdx2 (trophectoderm). Scale bars 20 µm. Control n = 3, FGF n = 3.

Source data are available on Github.

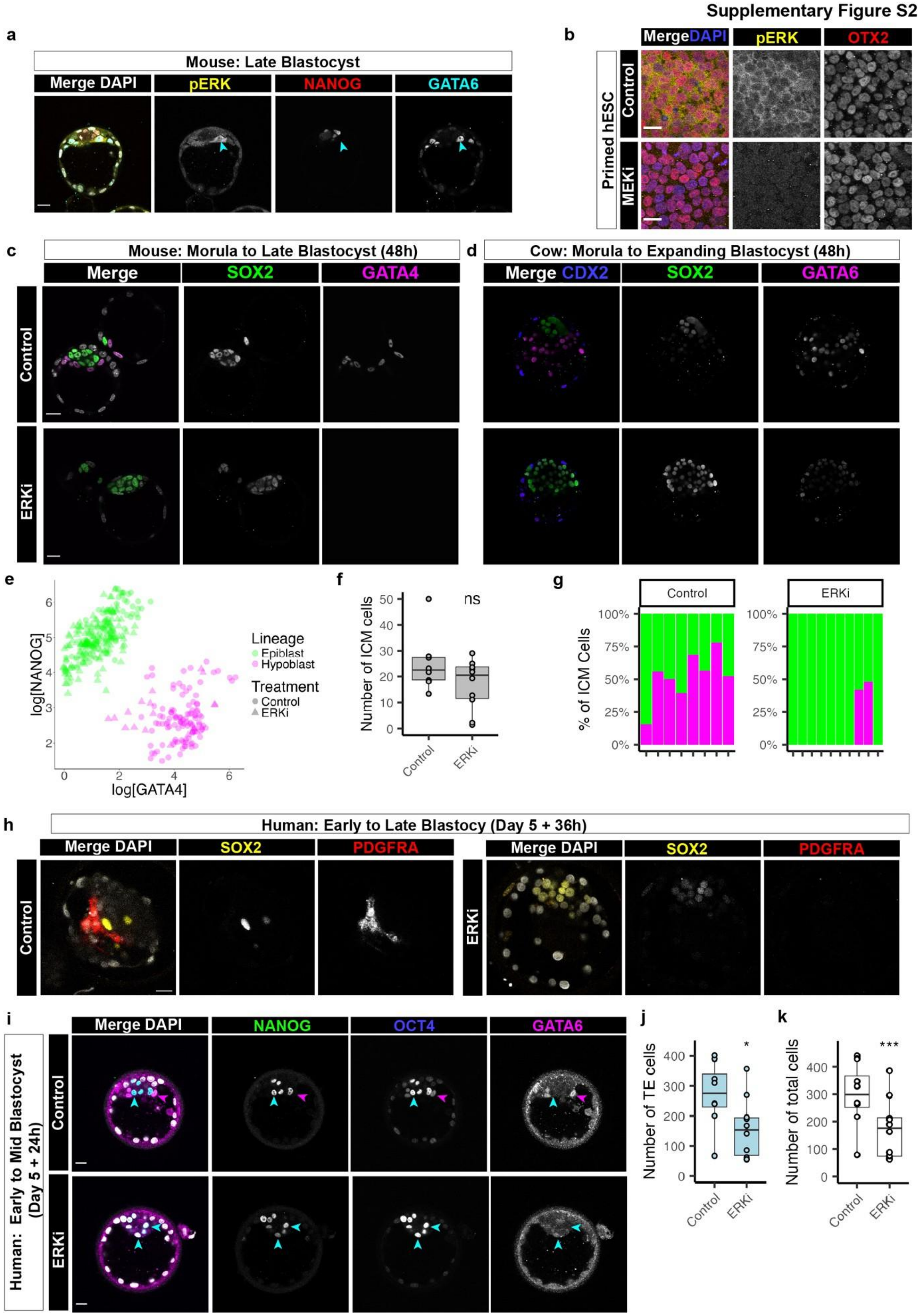

Supplementary Figure S2. Suppression of ERK signalling blocks hypoblast formation in the human blastocyst.

(a) Confocal images of mouse embryos immunofluorescently labelled for phosphorylated (p)-ERK, lineage markers Nanog (epiblast), Gata6 (hypoblast), and stained for nuclear DAPI. Cyan arrow indicates a hypoblast cell with high pERK levels. Scale bars 20μm. **(b)** Confocal images of human primed embryonic stem cells (ESC) treated for 24 hrs with either control media or following MEK-inhibition with PD0325901. Cells were immunofluorescently labelled for phosphorylated (p)-ERK and OTX2 (primed hESC marker) and stained for nuclear DAPI. Scale bars 20μm.

(c) Confocal images of mouse embryos following either ERKi or control treatment from day 2.5 morula stage for 48 hrs until day 4.5 late blastocyst stage. Mouse embryos were immunofluorescently labelled for lineage markers Sox2 (epiblast), Gata4 (hypoblast), and stained for nuclear DAPI. Scale bars 20μm. Control n = 5, ERKi n = 4.

(d) Confocal images of cow embryos following either ERKi or control treatment from day 7 morula stage for 48 hrs until day 9 blastocyst stage. Cow embryos were or immunofluorescently labelled for lineage markers Sox2 (epiblast), Gata6 (hypoblast), or Cdx2 (trophectoderm) and stained for nuclear DAPI. Scale bars 20μm. Control n = 3, ERKi n = 3.

(e) Scatter plot of NANOG and GATA4 nuclear fluorescence intensity of confocal images of human embryos in Fig. 2D. Hierarchical clustering was used to assign cell lineages. epiblast
($NANOG^{hi}GATA4^{lo}$) and hypoblast ($NANOG^{lo}GATA4^{hi}$)

(f) Boxplots showing the number of inner cell mass (ICM) cells (GATA3-) cells in human embryos cultured with and without ERKi. Boxplots represent the interquartile (IQR range), with the median shown as a central line; whiskers extend to lowest or highest value within 1.5*IQR; values for each embryo are shown as individual points. n.s. = $p > 0.05$, Two tailed t-test. Control n = 8, ERKi n = 10.

(g) Stacked bar charts showing the proportion of epiblast and hypoblast in the ICM of individual human embryos, grouped by treatment. Embryos are ordered left to right on the xaxis in ascending order of total number of cells. Control n = 8, ERKi n = 10.

(h) Confocal images of Day 6.5 human embryos immunofluorescently labelled for lineage markers SOX2 (epiblast), PDGFRA (hypoblast) and stained for nuclear DAPI. Human embryos were cultured in either ERKi or control media from Day 5 for 36 hours. Scale bars 20μm. Control n = 4, ERKi n = 4.

(i) Confocal images of Day 6.0 human embryos immunofluorescently labelled for lineage markers NANOG, OCT4 (epiblast) and GATA6 (hypoblast) and stained for nuclear DAPI.

Human embryos were cultured in either ERKi or control media from Day 5 for 24 hours. Pink and cyan arrowheads indicate hypoblast and epiblast cells, respectively. Scale bars 20μm. Control n = 3, ERKi n = 3.

(j-k) Boxplots showing **(j)** the number of trophectoderm (GATA3+) cells and **(k)** total cells in human embryos cultured with and without ERKi. Boxplots represent the interquartile (IQR range), with the median shown as a central line; whiskers extend to lowest or highest value within 1.5*IQR; values for each embryo are shown as individual points. * $p < 0.05$, *** $p < 0.001$, Two-tailed t-test. Control n = 8, ERKi n = 10. Across all species culture experiment were performed with an ERK inhibitor (5μM Ulixertinib) or volume matched DMSO control treatment.

Source data are available on Github.

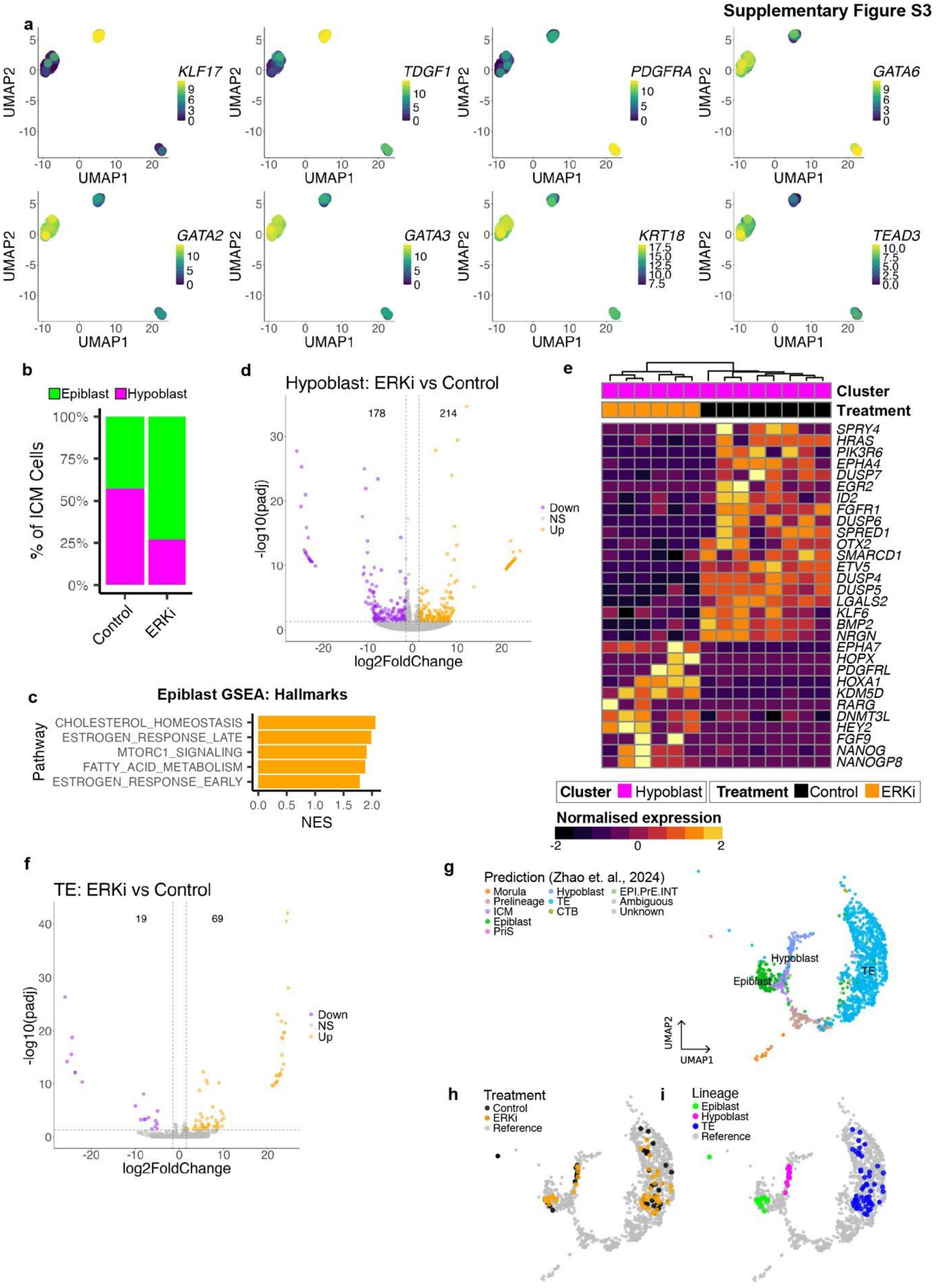

Supplementary Figure S3. ERKi of human embryos alters lineage specification and maintains naïve pluripotency characteristics.

(a) UMAP of single-cells from Day 6.5 human embryos cultured in ERKi or control medium, coloured by expression of epiblast (*KLF17, TDGF1*), hypoblast (*PDGFRA, GATA6*) or trophectoderm (*GATA2, GATA3, KRT18, TEAD3*) marker genes. Scales show log transformed normalised expression values.

(b) Stacked bar chart showing the proportion of epiblast and hypoblast cells in the single-cell RNAseq dataset (Fig. 3b), grouped by treatment.

(c) Top 5 gene set enrichment analysis (GSEA) of hallmark gene sets associated with differentially expressed genes in ERKi versus control cells in the epiblast cluster. Normalised enrichment score (NES) indicates gene sets are upregulated (orange).

(d-e) Volcano plot with genes significantly differentially expressed between ERKi treated and control cells within the **(d)** hypoblast or **(e)** trophectoderm (TE) cluster in (Fig. 3b). Significantly genes (DESeq2 padj < 0.05 and log2FC > 1.5) upregulated (orange) or downregulated (purple) in ERKi vs control. Adjusted p-values were calculated using the twosided Benjamini–Hochberg method to control for FDR across multiple tests.

(f) Hierarchical clustering and heatmap of selected significantly differentially expressed genes between ERKi treated and control cells within the hypoblast cluster. Normalized expression shown as z-score.

(g-i) UMAP integrating this study with a compiled human embryo reference dataset[7], coloured according to **(h)** lineage prediction using the reference tool and **(i)** DMSO-treated control (black) or ERKi (orange) cells from this study and cells from reference untreated control conditions (grey).

Source data are available on Github.

Supplementary Figure S4

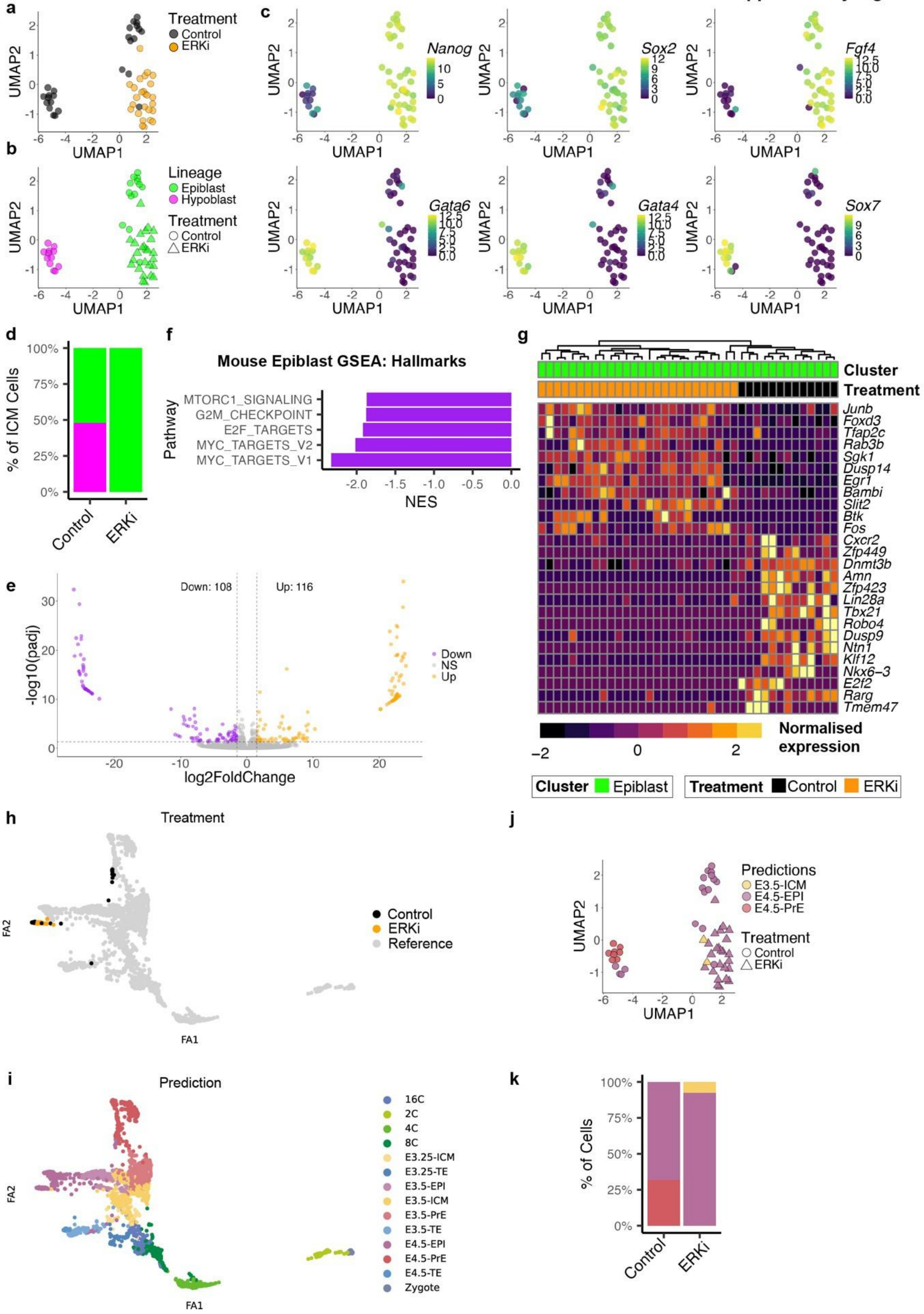

Supplementary Figure S4. Mouse single-cell transcriptomics reveals conserved ERK function in hypoblast specification but divergence in pluripotent epiblast lineage maintenance

(a) UMAP of single cells from Day 4.25 mouse embryos cultured in ERKi (orange) or control medium (black) from Day 3.25 for 24 hours.

(b) UMAP coloured according to mouse lineage identity.

(c) UMAP coloured by expression of mouse epiblast (*Nanog*, *Sox2*, *Fgf4*) or hypoblast (*Gata6*, *Gata4*, *Sox7*) marker genes. Scales show log transformed normalised expression values. **(d)** Stacked bar chart showing the proportion of mouse epiblast and hypoblast cells in the single-cell RNAseq dataset (Fig. 3b), grouped by treatment.

(e) Volcano plot with genes significantly differentially expressed between mouse ERKi treated and control cells within the epiblast cluster in (a). Genes significantly upregulated (orange) or downregulated (purple) in ERKi vs control cells (DESeq2 padj < 0.05 and log2FC > 1.5). Adjusted p-values were calculated using the two-sided Benjamini–Hochberg method to control for FDR across multiple tests.

(f) Top 5 gene set enrichment analysis (GSEA) of hallmark gene sets associated with differentially expressed genes in mouse ERKi versus control cells in the epiblast cluster. Normalised enrichment score (NES) indicates gene sets are downregulated (purple). **(g)** Hierarchical clustering and heatmap of selected significantly differentially expressed genes between mouse ERKi treated and control cells within the epiblast cluster. Normalized expression shown as z-score.

(h-i) UMAP integrating this study with a compiled mouse embryo reference dataset[8], coloured according to **(h)** dataset, this study (orange) and the reference (blue). **(i)** Lineage prediction using a deep learning-model[8].

(j) UMAP shown in (a) coloured by predictions from deep learning-model.

(k) Stacked bar chart showing lineage prediction of mouse cells from this study using a deep learning-model in control and ERKi conditions.

Source data are available on Github.

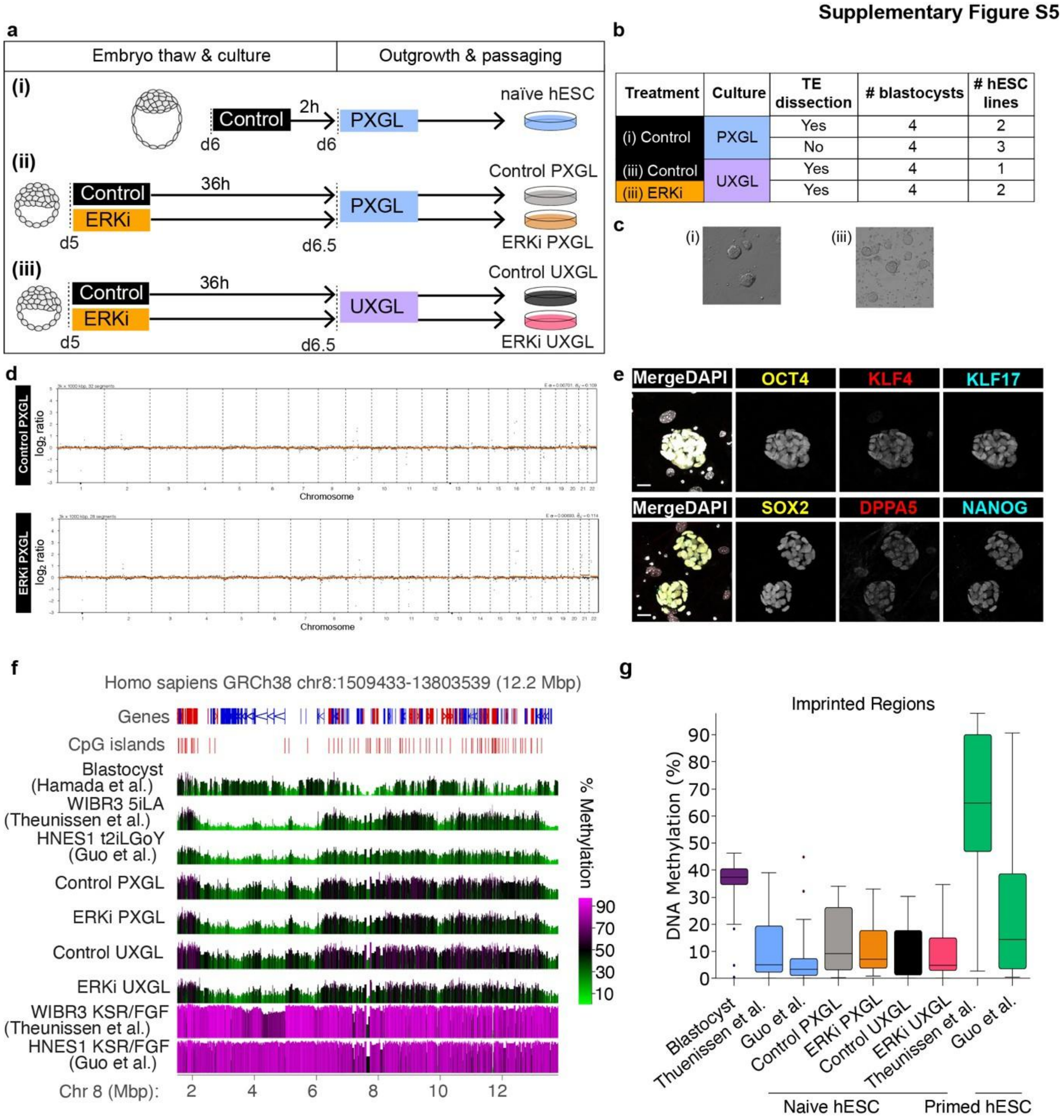

Supplementary Figure 5. Suppression of ERK signalling promotes pluripotent epiblast identity.

(a) Schematic of naïve hESC derivations. (i) Day 6 control in PXGL (ii-iii) Day 6.5 Control and ERKi embryo pre-treatment and derivation in (ii) PXGL (conditions in Fig. 4b-e) and (iii) UXGL.

(b) Table showing outcome of condition (i) and (iii) naïve ESC derivations. Condition (ii) shown in Fig. 4b.

(c) Representative phase-contrast image condition (i) naïve hESC line (n = 5) and condition (iii) ERKi UXGL naïve hESC (n = 2) lines. Condition (ii) shown in Fig. 4c.

(d) Representative low-pass whole genome sequencing followed by mapping to genomic DNA for karyotype analysis of hESC derived in PXGL media following either ERKi or control treatment. Representative of Control n = 2, ERKi n = 5.

(e) Confocal images of an ERKi UXGL naïve hESC line, immunofluorescently labelled for naïve (DPPA5, KL4, KLF17) and core (SOX2, OCT4, NANOG) pluripotency markers and stained for nuclear DAPI. Scale bars 20μm. Representative images Control UXGL n = 1, ERKi UXGL n = 2.

(f) Screenshot of DNA methylation in 100-CpG windows, across a 12Mbp region of chromosome 12 for blastocyst (n=1), primed hESC, naive hESC as well as the experimental conditions, Control PXGL (n=2), ERKi PXGL (n=3), Control UXGL (n=1) and ERKi UXGL (n=1).

(g) Boxplots showing DNA methylation of imprinted regions in blastocyst (n=1), primed hESC, naive hESC as well as the experimental conditions, Control PXGL (n=2), ERKi PXGL (n=3), Control UXGL (n=1) and ERKi UXGL (n=1).

Supplementary Data 1.

Differential gene expression analysis (DESeq2) comparing transcriptional differences between ERKi versus control cells, for epiblast, hypoblast and trophectoderm cells in human embryos.
This table is provided as an excel spreadsheet.

Supplementary Data 2.

Differential gene expression analysis (DESeq2) comparing transcriptional differences between ERKi versus control cells, for epiblast cells in mouse embryos.
This table is provided as an excel spreadsheet.

Supplementary Table 1.

List of primary and secondary antibodies used in this study.

Antibody	Host species	Company	Cat. No.	Dilution
**Primary**				
Anti-CDX2	Mouse	Biogenex	MU392A-UC	1:200
Anti-DPPA5	Rabbit	Sigma	D2569	1:250
Anti-GATA3	Rabbit	Abcam	ab199428	1:200
Anti-GATA4	Rat	eBioscience	14-9980-82	1:400
Anti-GATA6	Rabbit	Cell Signalling	5851	1:1000
Anti-GATA6	Rabbit	Santa Cruz	SC-9055	1:500
Anti-KLF4	Goat	R&D	AF3640	1:200
Anti-KLF17	Rabbit	Atlas	HPA024629	1:200 to 1:500
Anti-NANOG	Goat	R&D	AF1997	1:200
Anti-NANOG	Mouse	Invitrogen	MA1-017	1:100
Anti-OCT4	Mouse	Santa Cruz	SC-5279	1:250
Anti-OTX2	Goat	R&D	AF1979	1:500
Anti-SOX2	Rat	eBioscience	14-9811-82	1:100
Anti-PDGFRA	Rabbit	Abcam	Ab203491	1:500
Anti-pERK	Rabbit	Cell Signaling	4370	1:200
Anti-SOX17	Goat	R&D	AF1924	1:200
Anti-SUSD2	Mouse	Biolegend	327401	1:250
Anti-TFAP2C	Goat	R&D	AF5059	1:200
**Secondary**				
Anti-Mouse A488	Donkey	Invitrogen	A21202	1:300 to 1:500
Anti-Mouse A555	Donkey	Invitrogen	A32773	1:300

Anti-Mouse A647	Donkey	Invitrogen	A31571	1:300
Anti-Rabbit A488	Donkey	Invitrogen	A21206	1:300
Anti-Rabbit A555	Donkey	Invitrogen	A32794	1:300
Anti-Rabbit A555	Donkey	Invitrogen	A31572	1:500
Anti-Rabbit A594	Donkey	Invitrogen	A21207	1:500
Anti-Rabbit A647	Donkey	Invitrogen	A31573	1:500
Anti-Goat A555	Donkey	Invitrogen	A32816	1:300
Anti-Goat A594	Donkey	Invitrogen	A11058	1:500
Anti-Goat A647	Donkey	Invitrogen	A21447	1:500
Anti-Rat A488	Donkey	Invitrogen	A21208	1:500
Anti-Rat A647	Donkey	Invitrogen	A48272	1:300 to 1:500

Supplementary Table 2.

List of reagents used in the study.

Item	Supplier	Cat. No.
Human embryo culture		
Global media	CooperSurgical	LGGG-050
Human serum albumin (HSA)	CooperSurgical	GHSA-125
Vit Kit-Thaw	Fujifilm	90137-SO
G-TL	Vitrolife	10145
Quinn’s Advantage Embryo Thaw Kit	CooperSurgical	10542010

Mineral oil	CooperSurgical	ART-4008-5P
Recombinant human FGF4	R&D	235-F4-025
Heparin	Sigma	H3149-25KU
DMSO	Sigma	D2650
Ulixertinib (BVD-523)	Cambioscience	HY-15816-5mg
Embryo$^+$ slides	Vitrolife	16450
Guinea Pig Serum Complement	Merk	234395 - 5ML
Rabbit anti-mouse serum	Sigma	M5774 - 2ML
Rabbit anti-human serum	Sigma	H8765 - 2ML
Acid Tyrode's	Sigma	T1788
Glass capillaries	World precisions instruments	TW100F-6
Molecular biology		
SMART-Seq v4 Ultra Low Input RNA Kit for Sequencing	Takara	634890
SMART-Seq mRNA LP	Takara	634772 and 634768
Nextera XT DNA Library Preparation Kit	Illumina	FC-131-1024

Supplementary References

1 Li, H. & Durbin, R. Fast and accurate long-read alignment with Burrows-Wheeler transform. *Bioinformatics* **26**, 589-595, doi:10.1093/bioinformatics/btp698 (2010).

2 Scheinin, I. *et al.* DNA copy number analysis of fresh and formalin-fixed specimens by shallow whole-genome sequencing with identification and exclusion of problematic regions in the genome assembly. *Genome Res* **24**, 2022-2032, doi:10.1101/gr.175141.114 (2014).

3 Wamaitha, S. E. *et al.* IGF1-mediated human embryonic stem cell self-renewal recapitulates the embryonic niche. *Nat Commun* **11**, 764, doi:10.1038/s41467-02014629-x (2020).

4 Blakeley, P. *et al.* Defining the three cell lineages of the human blastocyst by singlecell RNA-seq. *Development* **142**, 3151-3165, doi:10.1242/dev.123547 (2015).
5 Petropoulos, S. *et al.* Single-Cell RNA-Seq Reveals Lineage and X Chromosome Dynamics in Human Preimplantation Embryos. *Cell* **165**, 1012-1026, doi:10.1016/j.cell.2016.03.023 (2016).
6 Yan, L. *et al.* Single-cell RNA-Seq profiling of human preimplantation embryos and embryonic stem cells. *Nat Struct Mol Biol* **20**, 1131-1139, doi:10.1038/nsmb.2660 (2013).
7 Zhao, C. *et al.* A comprehensive human embryo reference tool using single-cell RNAsequencing data. *Nat Methods* **22**, 193-206, doi:10.1038/s41592-024-02493-2 (2025).
8 Proks, M., Salehin, N. & Brickman, J. M. Deep learning-based models for preimplantation mouse and human embryos based on single-cell RNA sequencing. *Nat Methods* **22**, 207-216, doi:10.1038/s41592-024-02511-3 (2025).
