# Supplementary material for: Suppression of ERK signalling promotes pluripotent epiblast in the human blastocyst
Source: Nat Commun. 2025 Jul 28;16:6922. doi: 10.1038/s41467-025-61830-x (PMC12304225; doi:10.1038/s41467-025-61830-x)
Supplement: Supplementary file 2 — Reporting Summary [file 41467_2025_61830_MOESM2_ESM.pdf]

nature research

Corresponding author(s): Kathy Niakan
Last updated by author(s): June 8th 2025

Reporting Summary

Nature Research wishes to improve the reproducibility of the work that we publish. This form provides structure for consistency and transparency in reporting. For further information on Nature Research policies, see Authors & Referees and the Editorial Policy Checklist.

Statistics

For all statistical analyses, confirm that the following items are present in the figure legend, table legend, main text, or Methods section.

n/a	Confirmed	
☐	☒	The exact sample size (n) for each experimental group/condition, given as a discrete number and unit of measurement
☐	☒	A statement on whether measurements were taken from distinct samples or whether the same sample was measured repeatedly
☐	☒	The statistical test(s) used AND whether they are one- or two-sided<br>*Only common tests should be described solely by name; describe more complex techniques in the Methods section.*
☐	☒	A description of all covariates tested
☐	☒	A description of any assumptions or corrections, such as tests of normality and adjustment for multiple comparisons
☐	☒	A full description of the statistical parameters including central tendency (e.g. means) or other basic estimates (e.g. regression coefficient) AND variation (e.g. standard deviation) or associated estimates of uncertainty (e.g. confidence intervals)
☐	☒	For null hypothesis testing, the test statistic (e.g. F, t, r) with confidence intervals, effect sizes, degrees of freedom and P value noted<br>*Give P values as exact values whenever suitable.*
☒	☐	For Bayesian analysis, information on the choice of priors and Markov chain Monte Carlo settings
☒	☐	For hierarchical and complex designs, identification of the appropriate level for tests and full reporting of outcomes
☐	☒	Estimates of effect sizes (e.g. Cohen's d, Pearson's r), indicating how they were calculated

Our web collection on statistics for biologists contains articles on many of the points above.

Software and code

Policy information about availability of computer code

Data collection

Raw embryo scRNAseq and hESC RNAseq produced for this study have been deposited in the GEO database under accession codes GSE250613 [https://www.ncbi.nlm.nih.gov/geo/query/acc.cgi?acc=GSE250613], GSE250614 [https://submit.ncbi.nlm.nih.gov/geo/submission/update/?acc=GSE250614], GSE297052 [https://www.ncbi.nlm.nih.gov/geo/query/acc.cgi?acc=GSE297052], GSE297478 [https://www.ncbi.nlm.nih.gov/geo/query/acc.cgi?acc=GSE297478], GSE239843 [https://www.ncbi.nlm.nih.gov/geo/query/acc.cgi?acc=GSE239843]. Processed scRNAseq data is available at Zendo repository 15128175 [https://doi.org/10.5281/zenodo.15128175]. All PBAT-seq data generated in naïve hESCs are protected due to data privacy laws and are available upon request from the European Genome-phenome Archive EGAS50000001006 [https://ega-archive.org/datasets/EGAS50000001006]. Raw confocal microscopy images generated in this study are available at Figshare 28597145 [https://doi.org/10.6084/m9.figshare.28597145].
Previously published single-cell RNA-seq data from human embryos were downloaded from GEO accessions GSE36552 [https://www.ncbi.nlm.nih.gov/geo/query/acc.cgi?acc=GSE36552] and GSE66507 [https://www.ncbi.nlm.nih.gov/geo/query/acc.cgi?acc=GSE66507] and at EMBL-EBI ArrayExpress accession number E-MTAB-3929 [https://www.ebi.ac.uk/arrayexpress/experiments/E-MTAB-3929/]. Primed and naive hESC RNAseq datasets were downloaded from the ENA Browser https://www.ebi.ac.uk/ena/browser/home accessions PRJEB7132 [https://www.ebi.ac.uk/ena/browser/view/PRJEB7132], PRJNA522065 [https://www.ebi.ac.uk/ena/browser/view/PRJNA522065], PRJNA575370 [https://www.ebi.ac.uk/ena/browser/view/PRJNA575370], PRJEB12748 [https://www.ebi.ac.uk/ena/browser/view/PRJEB12748], and PRJEB47485 [https://www.ebi.ac.uk/ena/browser/view/PRJEB47485].

Data analysis

Human embryo scRNAseq: Technical replicate FASTQ raw sequencing files were merged and processed according to a modified nf-core/scrnaseq pipeline. Reads were trimmed to remove adapter sequences and aligned to the human GRCh38 reference genomes using STAR aligner. Transcript abundances, using the Ensembl gene annotations (versions 110 for human) were estimated using STAR quantmode, converted into a count matrix and further processed using scanpy. No computational doublet detection was performed. We excluded samples in which more than 30% of total reads aligning to the mitochondrial genome and fewer than 8,000 unique genes were detected.

hESC RNA-seq analysis: Technical replicate fastq files were merged and preprocessed using the nf-core/rnaseq pipeline 3.10.1 with Nextflow 22.10.1. Briefly, reads were trimmed for adaptors and quality using Trimgalore 0.6.7 and cutadapt 3.4. Trimmed reads were pseudoaligned to GRCh38 with Salmon 1.9.0.

Mouse embryo scRNAseq: Technical replicate FASTQ raw sequencing files were merged and processed according to a modified nf-core/scrnaseq pipeline. Reads were trimmed to remove adapter sequences and aligned to the mouse GRCm38 reference genomes using STAR aligner. Transcript abundances, using the Ensembl gene annotations (versions 102 for mouse) were estimated using STAR quantmode, converted into a count matrix and further processed using scanpy. No computational doublet detection was performed. We excluded samples in which more than 15% of total reads aligning to the mitochondrial genome.

Image analysis pipeline, code and source data for this paper are available on Github 15640445 [https://doi.org/10.5281/zenodo.15640445] and 1512875 [https://doi.org/10.5281/zenodo.15128175].

For manuscripts utilizing custom algorithms or software that are central to the research but not yet described in published literature, software must be made available to editors/reviewers. We strongly encourage code deposition in a community repository (e.g. GitHub). See the Nature Research guidelines for submitting code & software for further information.

Data

Policy information about availability of data

All manuscripts must include a data availability statement. This statement should provide the following information, where applicable:

- Accession codes, unique identifiers, or web links for publicly available datasets
- A list of figures that have associated raw data
- A description of any restrictions on data availability

Raw embryo scRNAseq and hESC RNAseq produced for this study have been deposited in the GEO database under accession codes GSE250613 [https://www.ncbi.nlm.nih.gov/geo/query/acc.cgi?acc=GSE250613], GSE250614 [https://submit.ncbi.nlm.nih.gov/geo/submission/update/?acc=GSE250614], GSE297052 [https://www.ncbi.nlm.nih.gov/geo/query/acc.cgi?acc=GSE297052], GSE297478 [https://www.ncbi.nlm.nih.gov/geo/query/acc.cgi?acc=GSE297478], GSE239843 [https://www.ncbi.nlm.nih.gov/geo/query/acc.cgi?acc=GSE239843]. Processed scRNAseq data is available at Zendo repository 15128175 [https://doi.org/10.5281/zenodo.15128175]. All PBAT-seq data generated in naïve hESCs are protected due to data privacy laws and are available upon request from the European Genome-phenome Archive EGAS50000001006 [https://ega-archive.org/datasets/EGAS50000001006]. Raw confocal microscopy images generated in this study are available at Figshare 28597145 [https://doi.org/10.6084/m9.figshare.28597145].

Field-specific reporting

Please select the one below that is the best fit for your research. If you are not sure, read the appropriate sections before making your selection.

☒ Life sciences ☐ Behavioural & social sciences ☐ Ecological, evolutionary & environmental sciences

For a reference copy of the document with all sections, see nature.com/documents/nr-reporting-summary-flat.pdf

Life sciences study design

All studies must disclose on these points even when the disclosure is negative.

Sample size	No statistical methods were used to predetermine sample size. Sample size was determined based on our previous experience and experience from other groups' work.<br>For human embryos:<br>Fogarty et al., Nature, 2017<br>Blakeley et al., Development, 2015<br>Niakan et al., Developmental Biology, 2013<br>For mouse embryos:<br>Hirate et al., Current Biology, 2013<br>Frum et al., Elife, 2018<br>For cow embryos:<br>Berg et al., Developmental Cell, 2011<br>Fouladi-Nashta et al., Reproductive Biomedicine Online, 2005
Data exclusions	No data were excluded from the study design.
Replication	Experiment were replicated at least three times and the data were reproducible in the different attempts of replication. All attempts at replication were successful.
Randomization	Samples were allocated randomly into experimental groups
Blinding	The investigators were not blinded to group allocation during experiments and outcome assessment due to the experimental design in which embryos had to undergo specific treatments.

Reporting for specific materials, systems and methods

We require information from authors about some types of materials, experimental systems and methods used in many studies. Here, indicate whether each material, system or method listed is relevant to your study. If you are not sure if a list item applies to your research, read the appropriate section before selecting a response.

Materials & experimental systems

n/a	Involved in the study
☐	☒ Antibodies
☐	☒ Eukaryotic cell lines
☒	☐ Palaeontology
☐	☒ Animals and other organisms
☐	☒ Human research participants
☒	☐ Clinical data

Methods

n/a	Involved in the study
☒	☐ ChIP-seq
☒	☐ Flow cytometry
☒	☐ MRI-based neuroimaging

Antibodies

Antibodies used

A list of all antibodies used, catalog number and concentrations is provided in Supplementary Table 3.
Anti-CDX2 Mouse Biogenex MU392AUC 1:200
Anti-DPPA5 Rabbit Sigma D2569 1:250
Anti-GATA3 Rabbit Abcam ab199428 1:200
Anti-GATA4 Rat eBioscience 14-9980-82 1:400
Anti-GATA6 Rabbit Cell Signalling 5851 1:1000
Anti-GATA6 Rabbit Santa Cruz SC-9055 1:500
Anti-KLF4 Goat R&D AF3640 1:200
Anti-KLF17 Rabbit Atlas HPA024629 1:200 to 1:500
Anti-NANOG Goat R&D AF1997 1:200
Anti-NANOG Mouse Invitrogen MA1-017 1:100
Anti-OCT4 Mouse Santa Cruz SC-5279 1:250
Anti-OTX2 Goat R&D AF1979 1:500
Anti-SOX2 Rat eBioscience 14-9811-82 1:100
Anti-PDGFRA Rabbit Abcam Ab203491 1:500
Anti-pERK Rabbit Cell Signaling 4370 1:200
Anti-SOX17 Goat R&D AF1924 1:200
Anti-SUSD2 Mouse Biolegend 327401 1:250
Anti-TFAP2C Goat R&D AF5059 1:200

Validation

Antibody validation was reported on the manufacturer's website.

Eukaryotic cell lines

Policy information about cell lines

Cell line source(s)

Human embryos were used to derive novel naive pluripotent stem cell lines which we are in the process of depositing into the UK Stem Cell Bank. In addition we used the H9 hESC line obtained under licence and SLA agreement with WiCell.
We also derived naive hESCs in either PXGL or UXGL culture conditions as described in the manuscript. The karyotype and sex chromosomes of the lines are:
Loke 1 UXGL DMSO2 (46, XY)
Loke 2 UXGL ULIX2 (46, XX)
Loke 3 UXGL (46, XY)
Loke 4 PXGL CS_1 (not determined)
Loke 5 PXGL CS_2 (not determined)
Loke 6 PXGL CS_6 (not determined)
Loke 7 PXGL CS_7 (not determined)
Loke 8 PXGL CS_8 (not determined)
Loke 9 PXGL DMSO_1 (46, XY)
Loke 10 PXGL DMSO_2 (46, XX)
Loke 11 PXGL Ulix_1 (46, XX)
Loke 12 PXGL Ulix_2 (46, XX)
Loke 13 PXGL Ulix_4 (46, XY)
Loke 14 PXGL Ulix_7 (46, XX)
Loke 15 PXGL Ulix_13 (46, XX)

Authentication

All of the hESC lines used have been tested by karyotpying, transcript and protein expression.
STA profiling was performed for the following lines:
H9
Loke 4 PXGL CS_1
Loke 5 PXGL CS_2
Loke 6 PXGL CS_6
Loke 7 PXGL CS_7
CLoke 8 PXGL S_8
Loke 9 PXGL DMSO_1
Loke 10 PXGL DMSO_2

	Loke 11 PXGL Ulix_1<br>Loke 12 PXGL Ulix_2<br>Loke 13 PXGL Ulix_4<br>Loke 14 PXGL Ulix_7<br>Loke 15 PXGL Ulix_13
Mycoplasma contamination	The cell lines were routinely tested for mycoplasma and were found to be negative.
Commonly misidentified lines (See ICLAC register)	N/A

Animals and other organisms

Policy information about studies involving animals; ARRIVE guidelines recommended for reporting animal research

Laboratory animals	Four- to eight-week-old (C57BL6×CBA) F1 female mice were super-ovulated using injection of 5 IU of pregnant mare serum gonadotrophin (PMSG; Sigma-Aldrich). Forty-eight hours after PMSG injection, 5 IU of human chorionic gonadotrophin (HCG; Sigma-Aldrich) was administered. Superovulated females were set up for mating with eight-week-old or older (C57BL6×CBA) F1 males. Mice were maintained on a 12h light–dark cycle, ambient temperature 19/22°C, and humidity 45/65%.
Wild animals	No wild animals were used in this study
Field-collected samples	Cow ovaries were obtained from the abattoir and transported to laboratory in PBS kept at 38°C.
Ethics oversight	Mouse research was performed in compliance with the UK Home Office Licence Number 70/8560.<br>The cow work was approved by the Ethics Committee at the Royal Veterinary College.

Note that full information on the approval of the study protocol must also be provided in the manuscript.

Human research participants

Policy information about studies involving human research participants

Population characteristics	This is not applicable as we used donated embryos surplus to IVF treatment. No sex or gender based analysis was performed on early human embryos from a variety of different backgrounds.
Recruitment	*Describe how participants were recruited. Outline any potential self-selection bias or other biases that may be present and how these are likely to impact results.*
Ethics oversight	This study was approved by the UK Human Fertilisation and Embryology Authority (HFEA): research licence numbers R0162, R0397, R0401 and R0152 and independently reviewed by the Health Research Authority's Research Ethics Committee IRAS projects 308099, 252286 and 272218

Note that full information on the approval of the study protocol must also be provided in the manuscript.
