# Supplementary material for: Suppression of ERK signalling promotes pluripotent epiblast in the human blastocyst
Source: Nat Commun. 2025 Jul 28;16:6922. doi: 10.1038/s41467-025-61830-x (PMC12304225; doi:10.1038/s41467-025-61830-x)
Supplement: Supplementary file 5 — Description of Additional Supplementary Files [file 41467_2025_61830_MOESM5_ESM.pdf]

Description of Additional Supplementary Files

Supplementary Data 1. Differential gene expression analysis (DESeq2) comparing transcriptional differences between ERKi versus control cells, for epiblast, hypoblast and trophectoderm cells in human embryos. This table is provided as an excel spreadsheet.

Supplementary Data 2. Differential gene expression analysis (DESeq2) comparing transcriptional differences between ERKi versus control cells, for epiblast cells in mouse embryos. This table is provided as an excel spreadsheet
